# Integration of BRAF V600E mutation with contrast-enhanced ultrasound parameters provides complementary diagnostic value for malignant thyroid nodules

**DOI:** 10.3389/fonc.2026.1729506

**Published:** 2026-05-08

**Authors:** Guihua Cui, Guanlin Wang, Chonghui Song, Ruimeng Tian, Kunwei Li, Zhihai Li, Ning Li

**Affiliations:** 1 Faculty of Life Science and Technology & The Affiliated Anning First People’s Hospital, Kunming University of Science and Technology, Kunming, Yunnan, China; 2 Faculty of Life Science and Technology, Kunming University of Science and Technology, Kunming, Yunnan, China; 3 Department of Ultrasound, The Affiliated Anning First People’s Hospital of Kunming University of Science and Technology, Kunming, Yunnan, China; 4 Imaging Department, Yunnan Maternal and Child Health Hospital, Kunming, Yunnan, China

**Keywords:** BRAF V600E mutation, contrast-enhanced ultrasound, diagnostic efficacy, thyroid nodules, time-intensity curve

## Abstract

**Objective:**

To evaluate the diagnostic efficacy of a combined model integrating the BRAF V600E mutation and quantitative contrast-enhanced ultrasound (CEUS) parameters for differentiating benign from malignant thyroid nodules(TNs).

**Design:**

A retrospective diagnostic study.

**Setting and participants:**

A total of 66 patients with TNs confirmed by surgical resection or core needle biopsy at our hospital between January 2023 and August 2025 were enrolled.

**Primary and secondary outcome measures:**

The primary outcome was the diagnostic performance, measured by the area under the receiver operating characteristic (ROC) curve, of the combined model compared to either the BRAF V600E mutation or CEUS parameter time to peak (TTP) alone. Secondary measures included the detection rate of BRAF V600E and the independent predictive value of each variable via multivariate logistic regression.

**Results:**

Among 66 patients (37 malignant, 29 benign), the BRAF V600E mutation was significantly more frequent in the malignant group (64.9% vs. 3.4%; P < 0.001). In a regression model containing only CEUS parameters, TTP was an independent predictor of malignancy (OR = 1.075, P = 0.013). However, with BRAF V600E added, TTP lost significance (P > 0.05), while BRAF V600E remained a significant predictor (OR = 38.380, P = 0.001). ROC analysis showed the combined model of TTP and BRAF V600E achieved the highest area under the curve (AUC) of 0.855, which was higher than using TTP alone (AUC = 0.681) or BRAF V600E alone (AUC = 0.807) (P < 0.05 for both comparisons).Notably, exploratory analysis suggested that CEUS parameters may provide complementary value in BRAF V600E-negative nodules, a clinically important subgroup where molecular testing alone is less informative. Compared with TI-RADS, the combined model achieved a higher specificity (64.9% vs. 24.1%) while maintaining comparable sensitivity (96.6% vs. 94.6%), indicating fewer false-positives.

**Conclusion:**

The BRAF V600E mutation is a strong independent predictor of thyroid nodule malignancy. Adding the quantitative CEUS parameter TTP to BRAF V600E yielded modest but significant diagnostic improvement vs BRAF V600E alone. Although validation in larger cohorts is needed, the integration of BRAF V600E mutation status with quantitative CEUS parameters may contribute to improved preoperative risk stratification, particularly in BRAF V600E-negative cases.

## 1 Introduction

Thyroid nodules (TNs), which are localized lesions arising from abnormal thyroid cell growth, are among the most common endocrine disorders. According to GLOBOCAN 2020 statistics, there were 586,000 new cases of thyroid cancer worldwide, ranking it ninth in incidence among all malignancies (1).Widespread use of ultrasonography has led to a high detection rate, reaching 20–68% in the general population (2–4). Although most TNs are benign, malignancy is confirmed in approximately 7–15% of cases, with papillary thyroid cancer (PTC) comprising 80–85% of these malignancies (3, 5–7). Accurate differentiation between benign and malignant nodules is therefore critical for determining appropriate treatment and improving patient prognosis (6, 8).

However, conventional ultrasonography has limitations in achieving precise diagnosis, including restricted resolution, the absence of typical features in some small PTCs, and operator-dependent subjectivity. These challenges have spurred the integration of molecular and advanced imaging tools into clinical practice. Among these, the BRAF V600E mutation is a well-established molecular event in PTC, with a global prevalence of 45–60% that rises notably in Asian populations—reaching 71–76% in East Asian countries according to a recent systematic review (9–11). This mutation drives constitutive activation of the MAPK pathway, promoting tumor proliferation and progression, and is strongly associated with aggressive clinicopathological features (10, 12). Despite its high specificity, sole reliance on BRAF V600E is limited by false-negative results in mutation-negative malignancies and potential false positives from assay artifacts (13).

Simultaneously, quantitative contrast-enhanced ultrasound (CEUS) allows assessment of microvascular perfusion in TNs. By analyzing time-intensity curve (TIC) parameters—such as peak intensity (pKI), time to peak (TTP), rise time (RT), and area under the curve (AUC)—CEUS can help distinguish malignant from benign lesions based on their vascular patterns (14–17). Nevertheless, diagnostic overlap between nodule types limits the standalone accuracy of CEUS parameters.

Therefore, this study aims to evaluate the complementary value of integrating BRAF V600E mutation status with CEUS quantitative parameters for differentiating benign from malignant TNs.

## 2 Methods

### 2.1 Subjects

This retrospective study was approved by the Medical Ethics Committee of the Affiliated Anning First People’s Hospital of Kunming University of Science and Technology (Approval No. 2023-032-01). Written informed consent was obtained from all individual participants.

Consecutive patients with TNs who underwent conventional ultrasound, CEUS, and pathological evaluation at our hospital between January 2023 and August 2025 were retrospectively screened from the institutional database. A stepwise selection process was applied based on the following criteria.

Inclusion criteria were: (a) completion of both conventional ultrasound and CEUS examinations; (b) availability of a definitive pathological diagnosis and BRAF V600E mutation status confirmed by ultrasound-guided fine-needle aspiration biopsy (FNAB) or surgical resection; (c) complete clinical and imaging data.

Exclusion criteria were: (a) poor-quality or incomplete imaging data; (b) pregnancy; (c) incomplete clinical records.

After applying the inclusion and exclusion criteria, a total of 66 patients were included in the final analysis, comprising 22 males and 44 females with a mean age of 43.25 years (range: 23–62 years).

Based on histopathological diagnosis, patients were divided into a benign group (n = 29) and a malignant group (n = 37). The benign group consisted of 4 inflammatory nodules, 12 nodular goiters, 8 Hashimoto’s thyroiditis-associated nodules, and 5 follicular adenomas. All malignant cases were pathologically confirmed as PTC.

### 2.2 Instruments and methods

#### 2.2.1 Conventional ultrasonography

Conventional ultrasonography was performed using the Mindray Resona R9S ultrasound system with an L14-3WU linear transducer (frequency range: 3–14 MHz). Patients were placed in the supine position with the neck extended to adequately expose the anterior cervical region. For each thyroid nodule, the following sonographic characteristics were recorded: location, number, maximum diameter, echogenicity, orientation (taller-than-wide shape), margin, and the presence of calcifications. All examinations were performed by a sonographer with more than 10 years of experience in thyroid ultrasound.

#### 2.2.2 Contrast-enhanced ultrasound and quantitative analysis

CEUS was performed using a Mindray Resona R9S ultrasound system equipped with a dedicated L9-3WU linear transducer (frequency range: 3–9 MHz). The contrast agent SonoVue (Bracco, Italy) was prepared.by adding 5 mL of normal saline to the vial and shaking vigorously to form a microbubble suspension. After selecting an imaging plane that clearly displayed the target lesion with abundant flow signals, the system was switched to contrast-specific imaging mode, and the probe was stabilized. Parameters including the mechanical index, depth, and gain were optimized. A bolus of 2.4 mL of the contrast suspension was rapidly injected via an antecubital vein, followed immediately by a 5 mL saline flush. Perfusion dynamics were observed for at least 3 minutes, and the enhancement intensity and patterns were recorded; the entire cine loop was saved for subsequent analysis.

Quantitative analysis was performed using the built-in quantification software of the Mindray system. For each thyroid nodule, two independent regions of interest (ROIs) were manually delineated: one within the lesion and another within the adjacent normal thyroid parenchyma on the same plane, following established methodologies. The ROI within the lesion was carefully placed on the solid component, avoiding cystic, necrotic, or calcified areas to ensure accurate perfusion assessment. The ROI size was adjusted to encompass the maximum solid area of the nodule, with consistent placement maintained across all measurements (17). ROI delineation was performed by two experienced sonographers, and any disagreements were resolved by consensus (18).The software automatically generated TICs) reflecting the wash-in and wash-out kinetics of the contrast agent. By comparing the TIC morphology and performing curve fitting, 14 quantitative parameters were derived. Including pKI, TTP, RT, AUC, among others.

To assess the reproducibility of these quantitative parameters, two experienced sonographers independently delineated the ROIs and measured all parameters, with neither aware of the other’s measurements nor the pathological diagnoses. Additionally, each sonographer repeated the measurements on the same images after a sufficient interval under blinded conditions to evaluate intra-observer consistency. The intraclass correlation coefficient (ICC) with a two-way random effects model for absolute agreement was used to evaluate both inter-observer and intra-observer consistency, with 95% confidence intervals (CIs) calculated. ICC values were interpreted as follows: < 0.50, poor; 0.50–0.74, moderate; 0.75–0.89, good; and ≥ 0.90, excellent. A two-tailed P < 0.05 was considered statistically significant for all ICC analyses.

All CEUS examinations were conducted by a sonographer with more than 10 years of experience in thyroid ultrasound. Acquired images were stored on hard drives. Two senior sonographers, blinded to the pathological results, independently analyzed all CEUS imaging data. In cases of disagreement, a third assessment was performed by an associate chief physician to reach a final consensus diagnosis based on comprehensive review of the image features.

#### 2.2.3 BRAF V600E mutation detection

The BRAF V600E mutation detection procedure was performed under strict aseptic conditions. Patients were placed in the supine position. Following standard skin disinfection, draping, and local anesthesia, a Bard 23G×50 mm biopsy needle was used to puncture the target lesion under real-time ultrasound guidance. Adequate specimens were obtained via multi-directional needle movement with suction. The aspirated material was immediately smeared and fixed on slides for cytopathological examination. Both the prepared slides and the needle rinse fluid were sent for pathological examination and BRAF V600E mutation analysis, respectively.

BRAF V600E mutation status was determined using a targeted next-generation sequencing (NGS) assay. DNA was extracted from the needle rinse fluid using a commercial DNA extraction kit (QIAamp^®^ DNA Mini Kit, Qiagen, Hilden, Germany). The NGS panel covered 30 thyroid-related genes, including BRAF (exons 11 and 15), and was designed to detect single nucleotide variants (SNVs) and small insertions/deletions (indels) in DNA, as well as gene fusions in RNA. Library preparation and sequencing were performed on the Illumina NextSeq550 or NovaSeq6000 platform (Illumina, San Diego, CA, USA).

The average sequencing depth was ≥3000×, with ≥85% of bases achieving ≥500× coverage and a target-on-target ratio ≥80%. The sequencing quality was high (Q30 ≥85%). The analytical sensitivity of the assay was approximately 10% for variants with a mutant allele frequency (MAF) ≥10% at 1000× depth, and 2% for variants with MAF ≥2% at 5000× depth. Quality control included positive and no-template controls in each run. Only samples meeting the predefined quality metrics (sufficient DNA yield, on-target ratio ≥80%, Q30 ≥85%) were included in the final analysis.

Variant interpretation followed the guidelines of the Association for Molecular Pathology (AMP), the American Society of Clinical Oncology (ASCO), and the College of American Pathologists (CAP), with variants classified into tiers based on clinical significance. The laboratory is accredited under CAP and ISO15189 standards.

Based on the genetic testing results, patients were ultimately categorized into two groups: the BRAF V600E mutation-positive group and the wild-type group.

### 2.3 Statistical analysis

Data analysis was performed using SPSS software (version 25.0,IBM Corp.Armonk, NY, USA). Continuous variables were presented as mean ± standard deviation (x̅ ± s) or median (with interquartile range), and comparisons between groups were conducted using the independent samples t-test or the Mann-Whitney U test, as appropriate. Categorical data were expressed as percentages (%) and analyzed using the χ² test or Fisher’s exact test.

To construct the diagnostic model, candidate variables were first screened via univariate analysis using the Mann-Whitney U test (for continuous variables) or the χ² test (for categorical variables). Variables with p-value < 0.05 in univariate analysis were considered candidates for inclusion in the multivariate logistic regression model. A stepwise forward conditional selection method was then applied to identify independent predictors of malignancy.

For the CEUS parameters, given the potential collinearity among them, we assessed multicollinearity using the variance inflation factor (VIF). Time to peak (TTP) was selected as the representative variable for inclusion in the multivariate model based on its clinical relevance and the strongest univariate association with malignancy, while ensuring that VIF values remained below 5 to avoid multicollinearity issues. The goodness-of-fit of the logistic regression model was evaluated using the Hosmer-Lemeshow test.

To assess diagnostic performance, ROC curves were plotted, and the AUC was calculated. The DeLong test was used to compare the AUC values to determine whether the combined model was superior to individual diagnostic indicators. To evaluate the real-world applicability, TI-RADS categories were analyzed using a cutoff of ≥4a to define a positive test for malignancy. The area under the ROC curve (AUC), sensitivity, specificity, positive predictive value (PPV), and negative predictive value (NPV) of TI-RADS were calculated, and its AUC was compared with that of the combined model using the DeLong test. A two-tailed *P*-value of < 0.05 was considered statistically significant for all tests.

## 3 Results

### 3.1 Surgical and pathological results

A total of 66 patients with 66 TNs were enrolled, including 22 males (33.3%) and 44 females (66.7%), with a mean age of 45.1 ± 9.5 years(range: 23–62 years). Based on BRAF V600E mutation status, 25 patients (37.9%) were mutation-positive (24 malignant, 1 benign) and 41 patients (62.1%) were wild-type.

All TNs were confirmed by surgical histopathology or fine-needle aspiration cytology. Of the 66 nodules,37 (56.1%) were and 29 (43.9%) were benign. All malignant nodules were PTC. The benign nodules consisted of 4 inflammatory nodules, 12 nodular goiters, 8 Hashimoto’s thyroiditis-associated nodules, and 5 follicular adenomas.

### 3.2 Comparison of baseline characteristics between benign and malignant thyroid nodules

No significant differences were observed between benign and malignant groups in terms of sex, age, or nodule location(**Table 1**). However, the detection rate of nodules ≤10 mm was significantly higher in the malignant group than in the benign group(81.1% vs. 55.2%; P < 0.05).Similarly, the BRAF V600E mutation rate was significantly higher in the malignant group (64.9% vs. 3.4%; P < 0.001).

**Table 1:** Characteristics comparison between malignant and benign thyroid nodules.

Characteristics		Benign (n =29 )	Malignant (n = 37)	*t/χ2*	*P*
Age		44.52 ± 9.76	45.51 ± 9.36	0.421	0.675
Sex				0.031	0.861
	males	10 (34.5%)	12 (32.4%)		
	females	19 (65.5%)	25 (67.6%)		
Location				0.055	0.973
	left	15 (51.8%)	20 (54.1%)		
	right	11 (37.9%)	13 (35.1%)		
	isthmic portion	3 (10.3%)	4 (10.8%)		
Diameter				5.167	0.023
	≤10mm	16 (55.2%)	30 (81.1%)		
	>10mm	13 (44.8%)	7 (18.9%)		
BRAF V600E gene				26.061	<0.001
	mutant	1 (3.4%)	24 (64.9%)		
	wild type	28 (96.6%)	13 (35.1%)		

**Figure 1:**
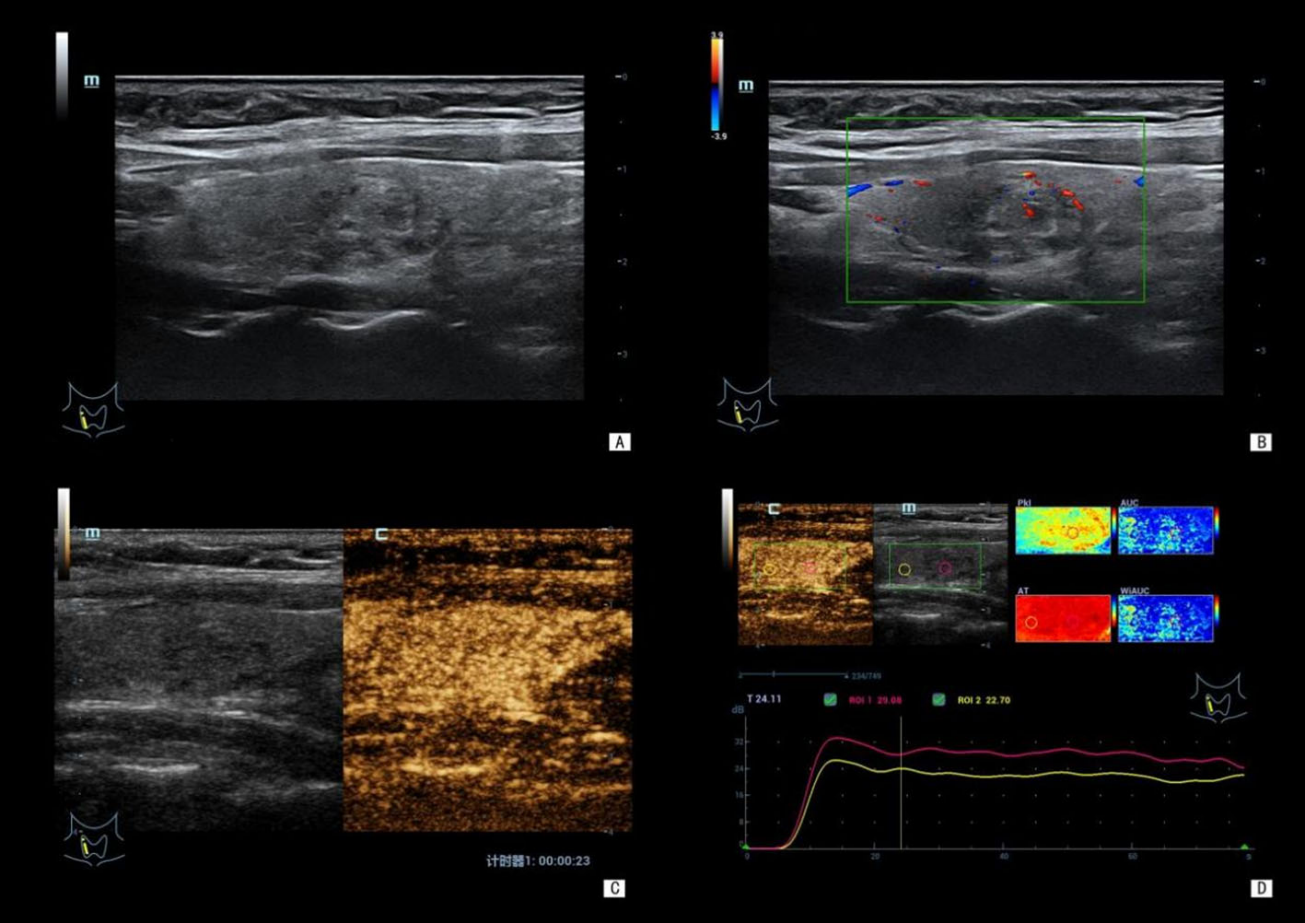
A 43-year-old female patient. Ultrasound images showed a nodule in the right thyroid lobe, measuring approximately 13 (left-right diameter) × 10 (anteroposterior diameter) × 18 (upper-lower diameter) mm. Postoperative pathology confirmed a benign nodule (nodular goiter), and BRAF V600E was wild-type. **(A)** Gray-scale ultrasound: the nodule showed horizontal growth, regular shape, clear margin, intact capsule, mixed cystic-solid echogenicity, and no internal microcalcifications. **(B)** Color Doppler ultrasound: punctate blood flow signals were visible within the nodule. **(C)** CEUS: the nodule exhibited homogeneous enhancement. **(D)** CEUS quantitative parameters: TTP: 14.18 s, AT: 6.93 s, mTIC: 25.33 dB. CEUS, contrast-enhanced ultrasound; BRAF V600E, BRAF V600E mutation; TTP, time to peak enhancement; AT, arrival time; mTIC, mean intensity.

**Figure 2:**
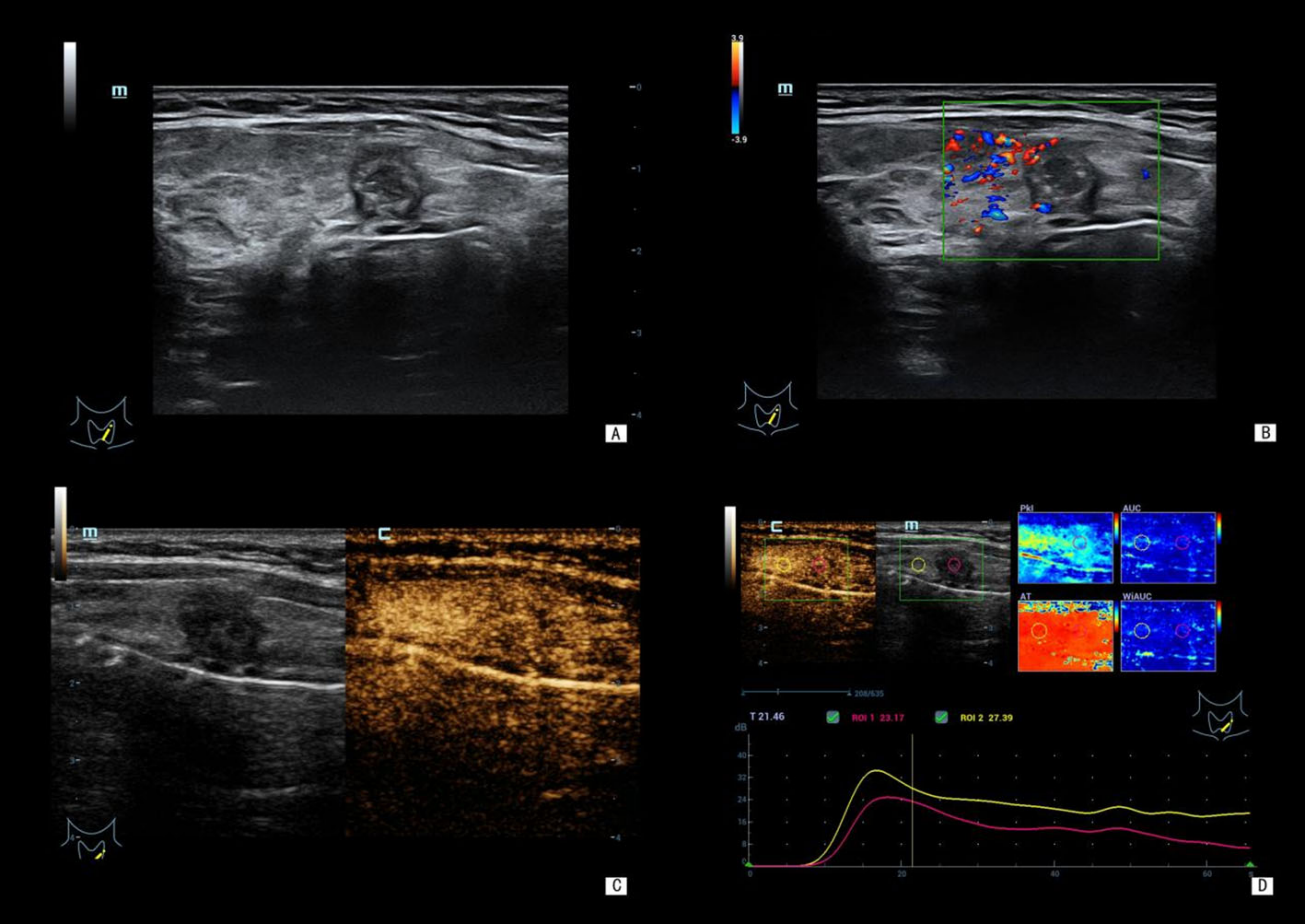
A 29-year-old female patient. Ultrasound images showed a nodule in the left thyroid lobe, measuring approximately 7 (left-right diameter) × 8 (anteroposterior diameter) × 10 (upper-lower diameter) mm. Postoperative pathology confirmed papillary thyroid carcinoma, and BRAF V600E was mutant. **(A)** Gray-scale ultrasound: the nodule showed vertical growth, ill-defined margin, solid hypoechoic texture, and visible microcalcification. **(B)** Color Doppler ultrasound: punctate-linear blood flow signal were visible within the nodule. **(C)** CEUS: the nodule exhibited inhomogeneous enhancement. **(D)** CEUS quantitative parameters: TTP: 18.21 s, AT: 10.45 s, mTIC: 11.94 dB. CEUS, contrast-enhanced ultrasound; BRAF V600E, BRAF V600E mutation; TTP, time to peak enhancement; AT, arrival time; mTIC, mean intensity.

Consistent with these findings, distinct sonographic features were observed between benign and malignant nodules (**Figures 1**, **2**). Malignant nodules typically exhibited irregular shape, hypoechogenicity, and a “fast wash-in and fast wash-out” contrast enhancement pattern. In contrast, benign nodules were more frequently characterized by regular shape, iso-/hyperechogenicity, and homogeneous contrast enhancement.

### 3.3 Comparison of quantitative CEUS parameters between benign and malignant thyroid nodules

Univariate analysis showed that the malignant group had significantly higher mean intensity (mTIC) and pKI values, and a significantly shorter TTP compared with the benign group (Z = -2.164, -2.248, -2.507, respectively; all P < 0.05). Parameters including AT, AUC, and AS showed marginal association with roup classification (0.05 ≤ P < 0.2), and were retained for further evaluation.

Before constructing the multivariate logistic regression model, candidate variables were screened for collinearity. A high correlation was observed between AS and pKI (r = 0.71, P < 0.001). Given this collinearity, the relatively smaller intergroup difference of AS compared with TTP and mTIC, and the lack of consensus on its diagnostic value in the literature. AS was excluded from the candidate variable pool to minimize redundancy and prioritize clinical relevance. Thus, five parameters—TTP, mTIC, pKI, AT, and AUC—were included in the subsequent multivariate logistic regression analysis to assess their independent predictive value. The remaining parameters showed no statistically significant differences between the two groups (**Table 2**).

**Table 2:** Comparison of quantitative analysis results of contrast-enhanced ultrasound in benign and malignant thyroid nodules.

Parameters	Benign (n =29 )	Malignant (n = 37)	*t/Z*	*P*
AREA	0.098 ± 0.057	0.086 ± 0.052	-1.282	0.200
mTIC	14.512 ± 6.143	17.446 ± 4.703	-2.164	**0.030**
pKI	26.485 ± 8.005	29.622 ± 5.580	-2.248	**0.025**
AUC	1277.055 ± 719.067	1452.719 ± 515.864	-1.738	0.082
AT	14.444 ± 8.741	10.612 ± 5.970	-1.848	0.065
WiAUC	240.436 ± 217.829	147.721 ± 42.995	-1.001	0.317
RT	16.528 ± 19.898	7.979 ± 4.858	-1.105	0.269
TTP	30.972 ± 22.078	18.567 ± 7.431	-2.507	**0.012**
WoAUC	1082.873 ± 702.119	1282.918 ± 504.006	1.347	0.183
AS	3.031 ± 2.184	3.718 ± 0.956	-1.848	0.065
DS	0.286 ± 0.325	0.236 ± 0.080	-0.045	0.964
SR	0.261 ± 0.655	0.080 ± 0.088	-1.051	0.293
FT	55.591 ± 29.186	62.851 ± 17.934	-1.176	0.240
mTT	67.350 ± 29.538	70.734 ± 17.774	-0.607	0.544

Bold font means P values are significant.

To assess the reliability of quantitative CEUS parameter measurements, intra- and inter-observer consistency were evaluated using the ICC. Inter-observer consistency analysis revealed excellent agreement for all 14 quantitative CEUS parameters between the two independent sonographers (ICC range: 0.808–0.928; all P < 0.001). The highest ICC values were observed for pKI (ICC = 0.928, 95% CI: 0.785–0.962) and TTP (ICC = 0.927, 95% CI: 0.826–0.962). Intra-observer consistency analysis also demonstrated excellent agreement for all parameters in repeated measurements by each sonographer after an interval of at least 1 week (ICC range: 0.863–0.928; all P < 0.001). The highest ICC values were observed for fall time (FT; ICC = 0.928, 95% CI: 0.827–0.971), mTIC (ICC = 0.926, 95% CI: 0.822–0.970, and wash-in area under the curve (WiAUC; ICC = 0.926, 95% CI: 0.825–0.970). These results confirm the high reproducibility and stability of our quantitative CEUS measurement protocol.

### 3.4 Candidate variable screening and collinearity diagnostics for multivariate logistic regression

Pearson correlation and VIF analyses were performed on the five candidate quantitative CEUS parameters (mTIC, pKI, AUC, TTP, AT) selected from univariate analysis.

A very strong positive correlation was observed between pKI and mTIC (r = 0.898, P < 0.001), with VIF values substantially exceeding the accepted threshold (8.962 and 8.336, respectively), indicating severe multicollinearity. A strong positive correlation was also found between mTIC and AUC (r = 0.811, P < 0.001), with a VIF of 3.377, suggesting moderate collinearity. A moderate positive correlation was observed between TTP and AT (r = 0.532, P < 0.001), with VIF values below 5, indicating no severe collinearity (**Table 3**).

**Table 3:** Results of collinearity verification of quantitative parameters of contrast-enhanced ultrasound.

Contrast-enhanced ultrasound parameters	Pearson corr. coeff. (max. with other params)	*P* value	VIF	Tolerance	Collinearity judgment
mTIC	*r* = 0.898 (strongly positive with pKI)	< 0.001	8.962	0.112	Serious collinearity
pKI	*r* = 0.898 (strongly positive with mTIC)	< 0.001	8.336	0.120	Serious collinearity
AUC	*r* = 0.811(strong positive with mTIC)	< 0.001	3.377	0.296	Moderate collinearity risk
TTP	*r* = 0.532 (moderate positive with AT)	< 0.001	1.825	0.548	No serious collinearity
AT	*r* = 0.532 (moderate positive with TTP)	< 0.001	1.825	0.458	No serious collinearity

Based on these results and clinical relevance, two variables were excluded from the final model.pKI was excluded due to severe collinearity with mTIC, as its diagnostic value was considered redundant. AUC was excluded primarily because of its weaker significance (higher P-value) in univariate analysis. The remaining three parameters—mTIC, TTP, and AT—passed the collinearity diagnostics (VIF < 5, tolerance > 0.2) and were included in the final multivariate logistic regression model (**Table 4**).

**Table 4:** The collinearity verification results of quantitative parameters of contrast-enhanced ultrasound after screening.

Contrast-enhanced ultrasound parameters	Pearson corr. coeff. (max. with other params)	*P*value	VIF	Tolerance	Collinearity judgment
mTIC	r = -0.380 (moderate negative with TTP)	0.002	1.168	0.856	Noncollinearity
TTP	r = 0.532 (moderate positive with AT)	< 0.001	1.562	0.640	No serious collinearity
AT	r = 0.532 (moderate positive with TTP)	< 0.001	1.395	0.717	No serious collinearity

### 3.5 Coefficients and collinearity statistics of CEUS parameters and BRAF V600E mutation

To construct a predictive model integrating quantitative CEUS parameters with the BRAF V600E mutation, we first assessed collinearity among the candidate predictors. Pearson correlation and VIF analyses showed that mTIC had a moderate negative correlation with TTP (r = -0.380, P = 0.002), while AT showed a moderate positive correlation with TTP (r = 0.532, P < 0.001). The BRAF V600E mutation was significantly positively correlated with TTP (r = 0.308, P = 0.012). All variables had VIF values below 5 and tolerance statistics greater than 0.2, indicating no severe multicollinearity. Therefore, all variables were retained for the subsequent multivariate logistic regression analysis (**Table 5**).

**Table 5:** The collinearity verification results of contrast-enhanced ultrasound parameters and BRAF V600E gene.

Variable	Pearson corr. coeff. (max. with other params)	*P* value	VIF	Tolerance	Collinearity judgment
mTIC	r = -0.380(moderate negative with TTP)	0.002	1.176	0.850	Noncollinearity
TTP	r = 0.532 (moderate positive with AT)	< 0.001	1.623	0.616	No serious collinearity
AT	r = 0.532 (moderate positive with TTP)	< 0.001	1.395	0.714	No serious collinearity
BRAF V600E gene	r = 0.308 (significant positive with TTP)	0.012	1.117	0.895	No serious collinearity

### 3.6 Multivariate logistic regression analysis of quantitative CEUS parameters for differentiating benign and malignant thyroid nodules

We performed multivariate logistic regression analysis using a forward stepwise method with TTP, mTIC, and AT as candidate variables. TTP was identified as the only independent predictor for differentiating benign from malignant TNs (OR = 1.075, 95% CI: 1.015–1.138, P < 0.05). Neither mTIC (P = 0.275) nor AT (P = 0.991) were retained in the final model (**Table 6**).

**Table 6:** Logistic regression analysis results of quantitative parameters of contrast-enhanced ultrasound in differentiating benign and malignant thyroid nodules.

Variable	*B* value	SE (b)	Wald χ²	*P* value	OR (95%CI)
TTP	0.072	0.029	6.140	0.013	1.075 (1.015~1.138)
mTIC	-0.003	0.004	0.563	0.275	0.997 (0.989~1.005)
AT	0.000	0.016	0.000	0.991	1.000 (0.969~1.032)

### 3.7 Logistic regression analysis of combined quantitative CEUS parameters and BRAF V600E mutation for differentiating benign and malignant thyroid nodules

We constructed a multivariate logistic regression model to evaluate the diagnostic value of combining TTP with the BRAF V600E mutation The BRAF V600E mutation was identified as an independent predictor for differentiating benign from malignant TNs (OR = 38.380, 95% CI: 4.583–321.427, P = 0.001).After adjusting for BRAF V600E mutation status, TTP was not an independent predictor in this combined model (P > 0.05)(**Table 7**).

**Table 7:** Logistic regression analysis contrast-enhanced ultrasound parameters combined with BRAF gene to identify benign and malignant thyroid nodules.

Variable	*B* value	SE (b)	Wald χ²	*P* value	OR (95%CI)
TTP	0.044	0.029	2.273	0.132	1.045 (0.987~1.107)
BRAF	3.648	1.084	11.316	0.001	38.380 (4.583~321.427)
Constant	-7.626	2.12	12.941	<0.001	0.001

### 3.8 Diagnostic performance assessed by ROC curve analysis

We plotted ROC curves to evaluate the diagnostic performance of each model. The AUC values for TTP alone, BRAF V600E mutation alone, and the combined model (TTP + BRAF V600E) were all significantly greater than 0.5, indicating their discriminative ability for differentiating benign from malignant TNs.

**Figure 3:**
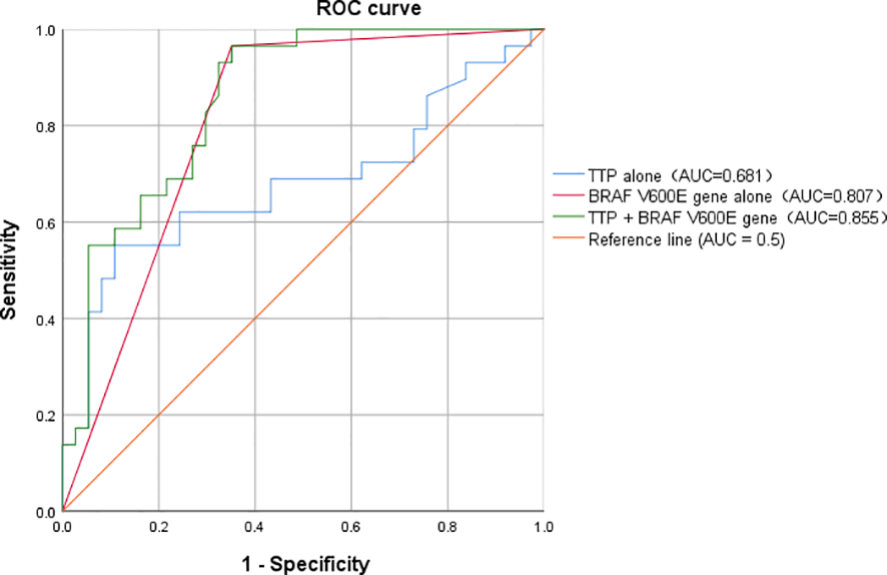
ROC curves of CEUS parameters and BRAF V600E mutation for differentiating benign from malignant thyroid nodules. The AUC for predicting malignant TNs was 0.681 for TTP alone, 0.807 for BRAF V600E alone, and 0.855 for the combined model(TTP + BRAF V600E). All AUC values were significantly greater than 0.5 (P < 0.05). TNs, thyroid nodules; ROC, receiver operating characteristic; TTP, time to peak enhancement; BRAF V600E, BRAF V600E mutation; AUC, area under the curve.

The combined model achieved the highest diagnostic efficacy, with an AUC of 0.855 (95% CI: 0.766–0.944, P < 0.001),which was higher than that of TTP alone (AUC = 0.681, 95% CI: 0.542–0.819, P = 0.012) and BRAF V600E mutation alone (AUC = 0.807, 95% CI: 0.700–0.914, P < 0.001) (**Table 8**; **Figure 3**). These results indicate that the combined model provides improved diagnostic accuracy for distinguishing benign from malignant TNs.

**Table 8:** ROC curve analysis results based on TTP and BRAF V600E gene.

Variable	AUC	95% CI	*P* value
TTP	0.681	0.542~0.819	0.012
BRAF V600E	0.807	0.700~0.914	<0.001
TTP+BRAF V600E	0.855	0.766~0.944	<0.001

AUC, area under the curve; CI, confidence interval. *P* values indicate whether the AUC is significantly greater than 0.5 (non-parametric test).

### 3.9 Exploratory analysis of BRAF V600E-negative nodules

To assess the potential diagnostic value of CEUS parameters in the absence of BRAF V600E mutation, we performed an exploratory analysis focusing on BRAF V600E-negative nodules. TTP values were compared between BRAF V600E-negative malignant nodules (n = 13) and BRAF V600E-negative benign nodules (n = 28) using the Mann-Whitney U test. Normality was assessed with the Shapiro-Wilk test, which indicated that TTP did not follow a normal distribution in either group (both P < 0.05)(**Table 9**).The median TTP was shorter in malignant nodules than in benign nodules (16.67 s, IQR: 14.79–22.95 s vs. 28.74 s, IQR: 17.74–34.21 s). However, the difference did not reach statistical significance (P = 0.057). Given the small number of BRAF V600E-negative malignant cases (n = 13), this exploratory analysis is underpowered to detect a statistically significant difference. Therefore, these findings should be interpreted as preliminary and warrant validation in larger cohorts.

**Table 9:** Comparison of TTP between benign and malignant nodules in the BRAF V600E-negative subgroup.

Group	n	Median TTP (s)	IQR (Q1–Q3)	Z	P value
Benign	28	28.74	17.74–34.21		
Malignant	13	16.67	14.79–22.95	-1.905	0.057

Mann-Whitney *U* test.

### 3.10 Comparison with TI-RADS

To assess the real-world applicability of the combined model, we compared its diagnostic performance with that of TI-RADS, a widely used ultrasound risk stratification system. Using a TI-RADS category ≥4a as the cutoff for malignancy, the area under the ROC curve (AUC) was 0.711 (95% CI: 0.587–0.835), with a sensitivity of 94.6%, specificity of 24.1%, positive predictive value (PPV) of 61.4%, and negative predictive value (NPV) of 77.8%. The combined model achieved a significantly higher AUC (0.855 vs. 0.711; P < 0.05, DeLong test). Using the optimal cutoff value of 0.2894 determined by the Youden index, the combined model achieved a sensitivity of 96.6%, specificity of 64.9%, PPV of 78.3%, and NPV of 95.0% (**Table 10**). Notably, the combined model maintained a high sensitivity while substantially improving specificity compared with TI-RADS (64.9% vs. 24.1%), indicating a reduction in false-positive predictions from 75.9% to 35.1%(**Table 10**).

**Table 10:** Comparison of diagnostic performance between the combined model and TI-RADS.

Diagnostic method	Cutoff	AUC (95% CI)	Sensitivity (%)	Specificity (%)	PPV (%)	NPV (%)
TI-RADS	≥4a	0.711 (0.587–0.835)	94.6	24.1	61.4	77.8
Combined model	0.29	0.855 (0.766–0.944)	96.6	64.9	78.3	95.0

TI-RADS, Thyroid Imaging Reporting and Data System; AUC, area under the curve; PPV, positive predictive value; NPV, negative predictive value. The optimal cutoff value for the combined model (0.29) was determined using the Youden index based on ROC curve analysis. DeLong test for AUC comparison between the combined model and TI-RADS: *P* < 0.05.

## 4 Discussion

The quantitative CEUS parameters demonstrated excellent intra- and inter-observer reproducibility (ICC > 0.80 for all parameters), confirming the reliability of our measurement protocol and minimizing potential bias from subjective ROI delineation.

Studies have identified the BRAF V600E gene as a significant oncogene in PTC. Our multivariate logistic regression analysis revealed that the BRAF V600E mutation is an independent predictor for differentiating benign from malignant TNs(OR = 38.38, P = 0.001). Notably, when only CEUS quantitative parameters were considered, TTP also emerged as an independent predictor (OR = 1.075, P = 0.013). However, after adjusting for BRAF V600E mutation status, TTP lost statistical significance(P > 0.05). This finding may reflect the dominant effect size of the BRAF V600E mutation, which is known to be highly specific for PTC, particularly given the relatively small sample size in our study. Rather than suggesting that molecular information supersedes imaging parameters, we interpret these results as indicating that BRAF V600E carries a particularly strong association with malignancy in our cohort, while TTP may still provide complementary value. The improvement in the area under the ROC curve (AUC) of the combined model compared with BRAF V600E alone (0.855 vs. 0.807; P < 0.05, DeLong test) may reflect the complementary value of TTP.

To further explore this potential complementary value, we performed an exploratory analysis focusing on BRAF V600E-negative nodules—a clinically important subgroup where molecular testing alone is less informative. In this subgroup, the median TTP was shorter in malignant nodules than in benign nodules (16.67 s vs. 28.74 s), although the difference did not reach statistical significance (P = 0.057). This finding suggests that CEUS parameters may offer complementary diagnostic information in cases where BRAF V600E mutation is absent. The lack of statistical significance is likely attributable to the small number of BRAF V600E-negative malignant cases (n = 13), which limited statistical power. Therefore, these results should be considered preliminary, and validation in larger cohorts of BRAF V600E-negative nodules is warranted to confirm the incremental value of CEUS parameters in this subgroup.

The improvement in AUC from 0.807 (BRAF V600E alone) to 0.855 (combined model), although modest in magnitude, was statistically significant (P < 0.05). In diagnostic testing, even a modest increase in AUC can have meaningful clinical implications when it translates into improved specificity and reduced false-positive rates. In our cohort, the combined model achieved a specificity of 64.9% compared with 24.1% for TI-RADS, representing a 40.8 percentage-point reduction in false-positive predictions. This improvement is clinically relevant as it could significantly reduce unnecessary fine-needle aspiration biopsies or surgeries in patients with benign nodules. Additionally, the high negative predictive value (95.0%) further supports the model’s utility in safely excluding malignancy. Therefore, while the absolute increase in AUC is modest, the corresponding improvements in clinically actionable metrics (specificity, false-positive rate, NPV) justify its potential value in preoperative risk stratification.

Our study differs from previous research in several important methodological and conceptual aspects. Zhu et al. investigated the diagnostic value of CEUS combined with BRAF V600E mutation in cytologically indeterminate TNs using six qualitative CEUS features assessed by visual interpretation (19); while their findings support the utility of this combined approach, qualitative assessment inherently carries subjectivity and may limit reproducibility. In contrast, we employed quantitative CEUS parameters derived from time-intensity curve analysis, with a specific focus on TTP, which offers greater objectivity and reproducibility. More recently, Hu et al. explored the role of quantitative CEUS parameters—particularly time-dependent metrics within both the nodule and surrounding parenchyma—as predictors of BRAF V600E mutation status, suggesting that CEUS may serve as a noninvasive tool for inferring molecular status (20). Our study addresses a distinct clinical question: integrating TTP with BRAF V600E mutation status to improve diagnostic accuracy. Rather than using CEUS to predict BRAF V600E mutation or relying on qualitative CEUS features, we constructed a combined predictive model and directly compared its diagnostic performance against each individual component using ROC curve analysis with DeLong testing. To our knowledge, this is one of the first studies to integrate a specific quantitative CEUS parameter (TTP) with BRAF V600E mutation status into a single predictive model and to directly quantify the incremental diagnostic value contributed by the combination. Our findings are consistent with previous reports (20, 21), supporting the concept that molecular markers may have greater predictive weight than imaging parameters in certain contexts, while highlighting the complementary role of CEUS parameters in mutation-negative cases.

When compared with TI-RADS, a widely used ultrasound risk stratification system, the combined model demonstrated superior diagnostic performance. The combined model achieved a significantly higher AUC (0.855 vs. 0.711; P < 0.05), with a marked improvement in specificity (64.9% vs. 24.1%) while maintaining comparable sensitivity (96.6% vs. 94.6%). This improvement translates into a substantial reduction in false-positive predictions (from 75.9% to 35.1%), highlighting the model’s potential to reduce unnecessary invasive procedures. The high negative predictive value (95.0%) further supports the model’s utility in safely excluding malignancy when the test result is negative. Even a recent large-scale study of 1,103 TI-RADS 4 nodules reported a clinical scoring model with an AUC of 0.943, highlighting the ongoing efforts to improve risk stratification (22).The limited specificity of TI-RADS observed in our cohort is consistent with previous reports (23). A previous study explored the combination of TI-RADS with other diagnostic tools. Wang et al. demonstrated that modified TI-RADS coupled with BRAF V600E mutation testing achieved an AUC of 0.945 (24). By integrating quantitative CEUS parameters with BRAF V600E mutation status, our combined model addresses this diagnostic gap. Our study extends previous findings by demonstrating that the combined model offers a more balanced trade-off between sensitivity and specificity compared with TI-RADS, particularly in the BRAF V600E-negative subgroup where molecular testing alone is less informative (19, 20). These findings suggest that the combined model may serve as a valuable adjunct to current clinical frameworks, especially when TI-RADS yields intermediate or suspicious results. However, validation in larger, prospective, multicenter cohorts is necessary before clinical implementation.

Numerous studies have confirmed the high specificity and high positive predictive value (PPV) of the BRAF V600E mutation in PTC (25). Similarly, CEUS has been shown to allow risk assessment through quantitative analysis of hemodynamic parameters, with TTP and PI being validated as independent predictors of malignancy (26, 27). The diagnostic synergy of BRAF V600E testing with other diagnostic modalities has been demonstrated in previous studies (19, 28). Our results are consistent with these findings and further suggest that the complementary value of CEUS parameters is most evident in the BRAF V600E-negative subgroup.

Several limitations of this study should be acknowledged. First, the retrospective design may introduce selection bias, despite the use of standardized inclusion criteria; moreover, the relatively small sample size limits the stability of our estimates, as reflected by the wide confidence intervals for the odds ratio of BRAF V600E (95% CI: 4.583–321.427).This also affected the exploratory analysis in the BRAF V600E-negative subgroup (n = 13 malignant cases), which was underpowered to detect a statistically significant difference. Furthermore, the study lacked internal validation and external validation, which increases the risk of overfitting and limits the generalizability of our findings. Second, the cohort included only PTC among malignant cases, precluding assessment of the model’s diagnostic performance in other thyroid malignancies. Given the high specificity of the BRAF V600E mutation for PTC, the proposed model should be interpreted within this histologic context. Third, as a single-center study, potential variations in genetic background and ultrasonographic practices across regions were not accounted for. Fourth, cytologically indeterminate nodules (Bethesda III-IV) were not included in the diagnostic analysis due to the lack of definitive histopathological diagnosis, precluding evaluation of the combined model in this clinically important subgroup and limiting the direct clinical applicability of our findings. Future studies should therefore focus on prospective, multicenter validation encompassing a broader spectrum of thyroid pathologies, including Bethesda III-IV nodules, while incorporating internal validation methods to further strengthen the robustness of the combined diagnostic approach.

## 5 Conclusion

The findings of this study confirm that the BRAF V600E mutation is a strong independent predictor for differentiating benign from malignant TNs. The diagnostic model combining the CEUS quantitative parameter TTP with BRAF V600E mutation achieved a modest but statistically significant improvement in diagnostic performance (AUC = 0.855) compared with BRAF V600E alone (AUC = 0.807).Notably, exploratory analysis suggested that CEUS parameters may provide complementary value in BRAF V600E-negative nodules, a clinically important subgroup where molecular testing alone is less informative—with a high NPV (95.0%) supporting the safe exclusion of malignancy. Although validation in larger, prospective, multicenter cohorts is needed, the integration of BRAF V600E mutation status with quantitative CEUS parameters may contribute to improved preoperative risk stratification, particularly in patients with BRAF V600E-negative nodules.

## Data Availability

The original contributions presented in the study are included in the article/**Supplementary Material**. Further inquiries can be directed to the corresponding author.
